# Altered ATP13A2/PARK9 Levels Influence α-Synuclein Accumulation in Neurons via Phagocytosis and Secretion in Glial Cells

**DOI:** 10.3390/cells14030163

**Published:** 2025-01-22

**Authors:** Taiji Tsunemi, Yuta Ishiguro, Asako Yoroisaka, Dou Feng, Nobutaka Hattori

**Affiliations:** Department of Neurology, Juntendo University School of Medicine, Tokyo 113-8421, Japan; yishigu@juntendo.ac.jp (Y.I.); a-yoroisaka@juntendo.ac.jp (A.Y.); d.feng.pv@juntendo.ac.jp (D.F.)

**Keywords:** Parkinson’s disease, ATP13A2, extracellular vesicles

## Abstract

(1) Background: Parkinson’s disease (PD) is characterized by the pathological accumulation of α-synuclein (α-syn) containing Lewy bodies (LBs) and Lewy neurites (LNs) within neurons. Growing evidence indicates that α-syn may propagate throughout the nervous system in a manner similar to prion-like transmission. Extracellular vesicles (EVs) may contribute to this pathway. We and others have reported that ATP13A2/PARK9 deficiency results in decreased EVs while its overexpression leads to increased EV generation. For analyzing EV-mediated α-syn secretion in neighboring neurons, we planned to alter Atp13a2 levels in vivo. (2) Methods: Three months after inoculating mouse α-syn fibrils into the striatum of *Atp13a2*-null and wild-type mice, we stained brain sections with anti-phosphorylated α-syn antibodies and then quantified LBs/LNs. We also examined the effect of increased levels of ATP13A2 by injecting lentivirus carrying human ATP13A2. Finally, we used cultured astrocytes and microglia for α-syn uptake and release, which were mediated by EVs. (3) Results: While LBs/LNs were formed in the entire brains, no significant difference was observed in LB/LN formation between Atp13a2-deficient and wild-type mice. Interestingly, the overexpression of ATP13A2 led to decreased LB/LN formation in the entire brains. Microglia and astrocytes released EVs more than neurons. EVs released from microglia and astrocytes contained more α-syn PFFs than those from neurons. (4) Conclusions: These results suggest that enhanced EV secretion by increased ATP13A2 levels attenuate the spreading of α-syn in brains, suggesting a protective role of ATP13A2 in α-synucleinopathies

## 1. Introduction

Parkinson’s disease (PD) is pathologically defined by the accumulation of Lewy bodies and Lewy neurites, primarily composed of alpha-synuclein (α-syn), a presynaptic protein involved in the development of both sporadic and familial PD [1]. Recent pathological and experimental studies have implicated that α-syn first accumulates in peripheral tissues or the central nervous system and then moves into each structure, spreading in the entire body [2]. The mechanism of α-syn transfer and spreading has been extensively studied for the past decade. Initially, from pathological observation in human autopsy cases, α-syn was expected to spread via retrograde axonal transport through the vagus nerve. While subsequent experimental studies using mice proved this transneuronal spread of α-syn from the gut to the CNS [3,4], some other experimental results failed to support this hypothesis [5,6]. Preformed fibrils (PFFs) inoculated with α-syn in the gastrointestinal tract spread within the brainstem but do not go further [5]. These results suggest an alternative pathway, which enables more widespread propagation in the central nervous system. One of the candidates includes extracellular vesicles (EVs), particularly exosomes, which are small extracellular vesicles that transport unnecessary materials out of cells and deliver messages to distant cells or tissues from host cells. EVs have attracted significant attention over the past decade due to their involvement in the pathomechanism of many neurodegenerative diseases, including PD [7]. We and others have reported that EVs carry α-syn and may regulate intracellular α-syn levels [8,9]. Kufor–Rakeb syndrome (KRS) is a rare neurodegenerative disorder primarily characterized by parkinsonism [10]. KRS is caused by the loss of function mutations in ATP13A2/PARK9, which encodes a lysosomal Type 5 P-type ATPase whose main function has been extensively explored but is still controversial. Initially, it was considered to serve cation homeostasis [11], and recent studies have demonstrated that it can serve as a lysosomal polyamine transporter [12] or lysosomal H^+^,K^+^-ATPase [13]. Dysfunctional ATP13A2 results in lysosomal dysfunction [14,15,16,17]. ATP13A2 is also localized to multivesicular bodies (MVBs) and plays a role in the formation of intraluminal vesicles (ILVs), which are released as extracellular vesicles (EVs) upon exiting the cell [8]. While decreased levels of ATP13A2 result in reduced EV production, enhanced expression increases it, suggesting the involvement of ATP13A2 in EV biogenesis. Both dysfunctional lysosomal proteolysis and impaired EV secretion due to ATP13A2 dysfunction can result in α-syn accumulation in cells, while the overexpression of ATP13A2 was shown to mitigate α-syn accumulation [8,18].

To examine the role of ATP13A2 in α-syn propagation in brains, we altered ATP13A2 expression levels in mouse brains. While the loss of ATP13A2 resulted in minimal alternation in α-syn propagation with decreased EV secretion, enhanced ATP13A2 expression increased α-syn accumulation in the injected area but decreased accumulation in areas lacking neuronal connection, accompanied by increased EV secretion. Further study revealed that injected α-syn PFFs were primarily taken up in microglia and astrocytes but only accumulated in neurons in a phosphorylated form. These results suggest that ATP13A2 levels affect α-syn propagation in mouse brains, at least in part, via the EV pathway.

## 2. Materials and Methods

### 2.1. Preparation of Recombinant A-Syn and Fibril Formation

Recombinant wild-type α-synuclein was purified as previously described [19]. In brief, a plasmid expressing human α-synuclein cDNA (pRK172) was transformed into *Escherichia coli* BL21 (DE3) cells, which were cultured, and protein expression was induced with 1 mM isopropyl-β-D-thiogalactopyranoside. The bacterial cell pellets were homogenized, and proteins were purified through several steps, including boiling at 100 °C for 10 min, Q-Sepharose ion-exchange chromatography, and ammonium sulfate precipitation. The α-syn proteins were dialyzed against 30 mM Tris–HCl buffer (pH 7.5) and cleared by centrifugation at 113,000× *g* for 20 min. Protein separation was performed by reverse-phase high-performance liquid chromatography (RP-HPLC) using an Aquapore RP300 column, and protein concentrations were measured. Purified human α-syn monomers (100 μM, 150 μL) were incubated at 37 °C in a shaking incubator at 1000 rpm in 50 mM Tris–HCl buffer containing 100 mM NaCl (pH 8.0) for 7 days. Fibril formation was monitored by turbidity measurements at OD600. After 7 days, α-syn fibrils were pelleted by centrifugation at 100,000× *g* for 30 min, resuspended in PBS, and sonicated for 3 min to generate α-syn preformed fibrils (PFFs). The formation of PFFs was confirmed using transmission electron microscopy.

### 2.2. Mice and Protocols for A-Syn PFF Injection into Mouse Brains

**Figure 1:**
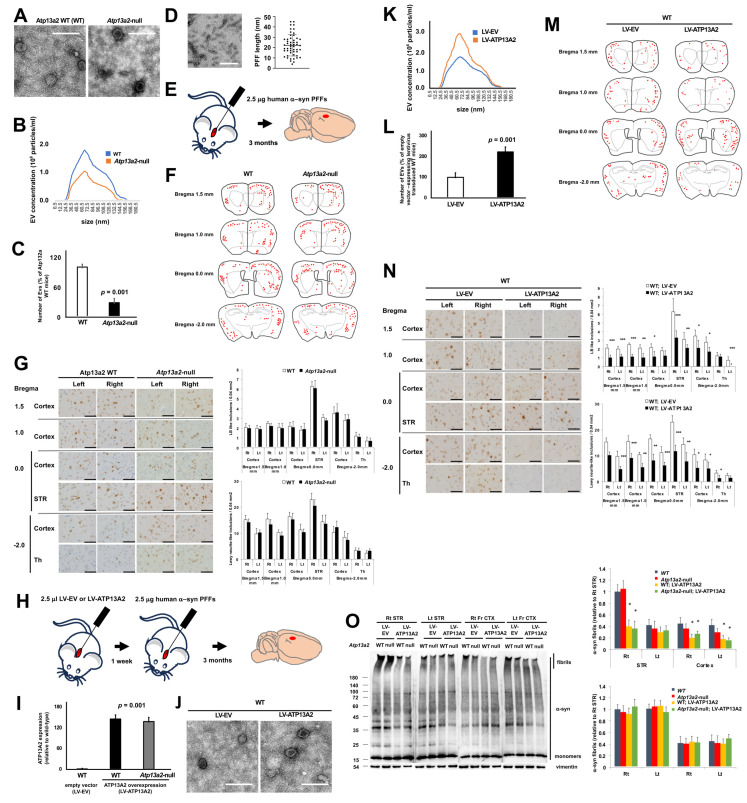
Alternations in ATP13A2 levels affect α-synuclein accumulation and propagation in mouse brains. (**A**) Electron microscopic analysis of EVs isolated from the brains of *Atp13a2* wild-type (WT) (*Atp13a2*^+/+^) and *Atp13a2*-null (*Atp13a2*^−/−^) mice. (**B**) Representative results of nanoparticle tracking analysis [21] of EVs isolated from the brains of WT and *Atp13a2*-null mice. (**C**) The number of EVs isolated from WT and *Atp13a2*-null mice (n = 3; *p* = 0.001). (**D**) A representative image of sonicated human alpha synuclein (α-syn) preformed fibrils (PFFs). (**E**) A diagram of the procedure of the first mouse experiment. The 2.5 µL (1.0 µg/µL) α-syn PFFs were injected into the right STR of WT and *Atp13a2*-null mice. Three months later, the mice were sacrificed for immunohistochemical (IHC) and biochemical analyses. (**F**) The distribution of Lewy body (LB)- (red circle) and Lewy neurite (LN)- (red line) like inclusions. The four sections were cut 1.5 mm anterior to the bregma (top), 1.0 mm anterior to the bregma (second from the top), at the bregma (second from the bottom), and 2.0 mm posterior to the bregma (bottom) from WT and *Atp13a2*-null mice, three months after human α-syn PFF inoculation. (**G**) Left: representative images of each region from WT and *Atp13a2*-null mice three months after human PFF inoculation (right). Six regions were selected for imaging from four sections. These regions included the cortex from all four sections, the STR from the section at the bregma, and the thalamus from the section 2.0 mm posterior to the bregma. The figure was assembled by combining these images. Right: quantitative analysis of LB- (upper) and LN- (bottom) like inclusions. Images were captured at a 400× original magnification, with 10 randomly selected pictures from each target region, and data were collected from 5 to 7 animals per group. STR stands for the striatum, and Th stands for the thalamus. (**H**) A diagram of the procedure of the second mouse experiment. Lentivirus carrying empty vectors (WT; LV-EV) or carrying human ATP13A2 (LV-ATP13A2) was injected into the right STR, one week prior to α-syn PFF inoculation. After three months, these mice were subjected to IHC analysis. (**I**) Results of quantitative reverse transcription polymerase chain reaction (RT-qPCR) analysis of human ATP13A2 mRNA expression in WT mice infected with LV-EV (WT; LV-EV), WT mice infected with LV-ATP13A2 (WT; LV-ATP13A2), and *Atp13a2*-null mice infected with LV-ATP13A2 (*Atp13a2*-null; LV-ATP13A2) (n = 3; *p* = 0.001). (**J**) Electron microscopic analysis of EVs isolated from brains of WT; LV-EV and WT; LT-ATP13A2 mice. (**K**) Representative results of nanoparticle tracking analysis (NTA) of EVs isolated from brains of WT; LV-EV and WT; LT-ATP13A2 mice. (**L**) The number of EVs isolated from WT; LV-EV and WT; LT-ATP13A2 mice (n = 3; *p* = 0.001). (**M**) The distribution of LB- (red circle) and LN- (red line) like inclusions in WT; LV-EV and WT; LT-ATP13A2 mice three months after human PFF inoculation. (**N**) Left: representative images of each region from the WT; LV-EV and WT; LT-ATP13A2 mice three months after human PFF inoculation. Right: quantitative analysis of LB- (upper) and LN- (bottom) like inclusions in 10 randomly selected pictures from each target region (n = 5–7; * *p* < 0.03; ** *p* = 0.01; *** *p* = 0.001). (**O**) Immunoblot analysis of spreading of PFFs in mouse brains taken from WT; LT-EV, *Atp13a2*-null; LV-EV, WT; LV-ATP13A2, and *Atp13a2*-null; LV-ATP13A2 mice. Two months after the injection, the mice were sacrificed, and the brains were taken out. After conducting protein extraction, samples from the right STR, left STR, right frontal cortex, and left frontal cortex were subjected to SDS PAGE followed by immunoblotting. Left: representative image of immunoblotting with anti-α-syn (C-20) and anti-Vimentin. Right: densiometric analysis of immunoblotting. Vimentin was used for normalization control (n = 3; * *p* = 0.01). CTX stands for the cortex. Values are mean ± SEM. Scale bars represent 100 nm for (**A**,**D**,**J**) and 100 μm for (**G**,**N**). The mean and SD were calculated from 5 to 7 mice per group. Data were analyzed by the Student *t*-test (n = 3, (**C**,**I**,**L**); n = 5–7, (**G**,**N**)) or one-way ANOVA followed by Tukey’s multiple comparisons test (n = 5–7, (**O**)).

**Figure 2:**
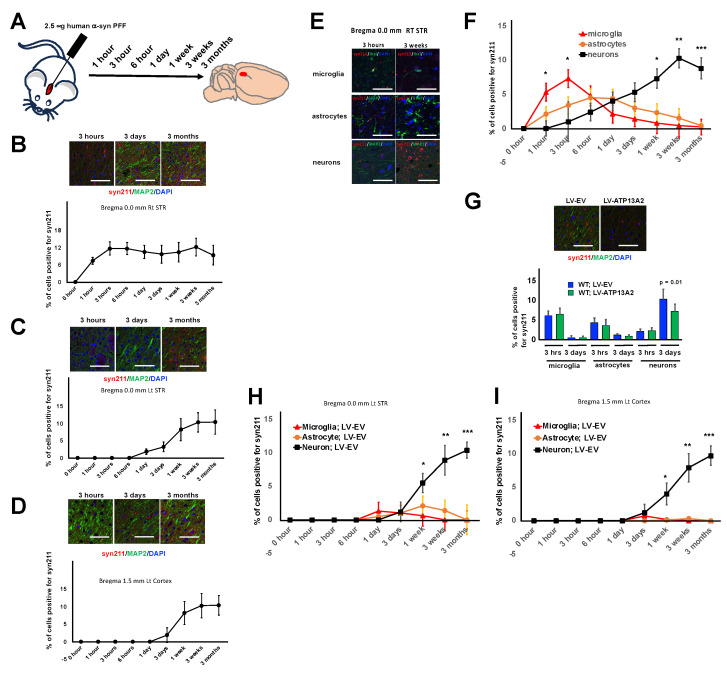
Inoculated α-syn PFFs were preferentially taken up by glial cells. (**A**) A diagram of the procedure of the third mouse experiment. The mice were perfused at 1, 3, and 6 h, as well as 1 day, 1 week, 3 weeks, and 3 months, post-inoculation of α-syn PFFs. (**B**) Top: representative images of α-syn PFF uptake in the section 0.0 mm from the bregma in the right STR 3 h, 3 days, and 3 weeks after α-syn PFF inoculation. Bottom: the change in the percentage of cells positive for the α-syn antibody Syn 211 in the section 0.0 mm from the bregma in the right STR after α-syn PFF injection (n = 5). (**C**) Top: representative images of α-syn PFF uptake in the section 0.0 mm from the bregma in the left STR 3 h, 3 days, and 3 weeks after α-syn PFF inoculation. Bottom: the change in the percentage of cells positive for the α-syn antibody Syn 211 in the section 0.0 mm from the bregma in the left STR after α-syn PFF injection (n = 5). (**D**) Top: representative images of α-syn PFF uptake in the section 0.0 mm from the bregma in the left cortex 3 h, 3 days, and 3 weeks after α-syn PFF inoculation. Bottom: the change in the percentage of cells positive for the α-syn antibody Syn 211 in the section 0.0 mm from the bregma in the left cortex after α-syn PFF injection (n = 5). (**E**) Representative images of α-syn PFF uptake in neurons, astrocytes, and microglia in the section 0.0 mm from the bregma in the right STR 3 h and 3 weeks after inoculation. (**F**) The change in the percentage of cells positive for the α-syn antibody syn 211 in neurons, astrocytes, and microglia in the section 0.0 mm from the bregma in the right STR (n = 5; * *p* = 0.01; ** *p* = 0.003; *** *p* = 0.001). (**G**) Top: representative images of α-syn PFF uptake in the section 0.0 mm from the bregma in the right STR from WT; LV-EV and WT; LV-ATP13A2 mice 3 h and 3 weeks after α-syn PFF inoculation. Bottom: the effect of ATP13A2 overexpression on α-syn PFF accumulation in neurons, astrocytes, and microglia in the section 0.0 mm from the bregma in the right STR (n = 5; *p* = 0.01). (**H**) The change in the percentage of cells positive for the α-syn antibody syn 211 in neurons, astrocytes, and microglia in the section 0.0 mm from the bregma in the left STR from WT; LV-EV and WT; LV-ATP13A2 mice (n = 5; * *p* = 0.04; ** *p* = 0.003; *** *p* = 0.001). (**I**) The change in the percentage of cells positive for the α-syn antibody syn 211 in neurons, astrocytes, and microglia in the section 1.5 mm from the bregma in the left cortex from WT; LV-EV and WT; LV-ATP13A2 mice (n = 5; * *p* = 0.01; ** *p* = 0.003; *** *p* = 0.001). Values are the mean ± SEM. Scale bars represent 100 µm for (**B**,**C**,**D**,**E**,**G**). The mean and SD were calculated from triplicate samples from two independent experiments. Data were analyzed by the Student *t*-test (n = 5, (**G**)) and one-way ANOVA followed by Tukey’s multiple comparisons test (n = 5–7, (**F**,**H**,**I**)).

*Atp13a2*-null mice were described previously [19]. Three different mouse experiments were conducted. First, to assess the effect of ATP13A2 deficiency, 2.5 µL (1.0 µg/µL) α-syn PFFs were injected into the right striatum (STR) of 2-to-3-month-old Atp13a2-null and wild-type (WT) mice, as previously described [20]. Three months later, the mice were sacrificed for immunohistochemical (IHC) and biochemical analyses (Figure 1E). In the second experiment, to assess the effect of ATP13A2 overexpression, lentivirus carrying human ATP13A2 (LV-ATP13A2) or lentivirus carrying an empty vector (LV-EV) was injected into the right STR at a multiplicity of infection of 1, one week prior to PFF inoculation. After three months, these mice were subjected to IHC analysis (Figure 1H). Finally, to analyze differences in α-syn PFF uptake among cell types, the mice were perfused at 1, 3, and 6 h, as well as 1 day, 1 week, 3 weeks, and 3 months post-inoculation of α-syn PFFs (Figure 2A). In each experiment, 5 to 7 mice per group were used.

### 2.3. Plasmids

Human ATP13A2 lentivirus was generously provided by Christopher Rochet. Lentiviruses containing short hairpin RNA (shRNA) plasmids targeting human ATP13A2, as well as scrambled controls, were purchased from Open Biosystems (GE Healthcare, Chicago, IL, USA). The virus titers were measured using the HIV-1 p24 Antigen ELISA kit (Zeptometrix, New York, NY, USA).

### 2.4. Injection of A-Syn PFFs

After making human α-syn PFFs by shaking, sonicated PFFs (2.5 μg/2.5 μL) were injected into the right STR (A-P: 0.2 mm; M-L + 2.3 mm; D-V: −2.6 mm, from the bregma) of the mice ranging between 2 and 3 months of age at a rate of 0.1 μL per minute. At various pre-determined time points, mice were anaesthetized with an isoflurane/oxygen/nitrogen mixture and either decapitated for biochemical analysis or perfused with PBS followed by 4% PFA in PBS.

### 2.5. EV Isolation and Nanoparticle Tracking Analysis

EVs were purified from cultured media through successive ultracentrifugation steps, as described previously [8]. Briefly, pre-conditioned media, free from EVs, were prepared by ultracentrifugation at 110,000× *g* overnight. After washing with phosphate-buffered saline (PBS), the cells were cultured in these pre-conditioned media for 24 h. EVs were isolated from the media using a standard differential centrifugation protocol (200× *g* for 5 min, 1200× *g* for 10 min, and 16,500× *g* for 30 min), followed by ultracentrifugation at 110,000× *g* for 60 min. EVs were isolated from mouse brains; this was conducted following the protocol in [22]. Briefly, the brain tissue was finely chopped into small pieces using a pre-warmed papain solution, which consisted of Hibernate A supplemented with 20 U/mL of papain. The fragments were then transferred into a tube containing papain solution and incubated at 37 °C for 15 min. Enzymatic digestion was halted by adding ice-cold papain inhibitor solution containing 5 μg/mL LAP, 1 mM PMSF, and 1 μM E64 in Hibernate A. The tissue was further dissociated by gentle pipetting and subsequently subjected to centrifugation at 300× *g* for 10 min at 4 °C. The resulting supernatant was passed through a 40 μm cell strainer to eliminate large debris, and the filtrate was further homogenized by passing it through a 26-gauge needle. The sample then underwent a second round of centrifugation at 2000× *g* for 20 min. After this spin, the supernatant was transferred to a fresh tube and subjected to ultracentrifugation at 100,000× *g* for 60 min. Following ultracentrifugation, the supernatant was discarded. The EVs were then washed in PBS and pelleted by centrifugation at 110,000× *g* for 60 min. Finally, the EV pellet was resuspended in PBS. EV analysis was performed using the NanoSight LM10 system (Malvern Panalytical Ltd., Malvern, UK), equipped with a 405 nm laser and a high-sensitivity digital camera system (OrcaFlash2.8, Hamamatsu C11440, Hamamatsu Photonics, Hamamatsu, Japan). Samples were introduced and recorded for 1 min under continuous flow, controlled by a script-based system with a NanoSight syringe pump. Videos were analyzed using NTA software (v2.3).

### 2.6. Alpha-Synuclein Detection

Alpha-synuclein enzyme-linked immunosorbent assay (ELISA) was performed as previously described [8,20].

### 2.7. Immunohistochemistry

After α-syn PFF injection, at the suggested time, the mice were perfused with PBS followed by 4% paraformaldehyde (PFA) in PBS for fixation. After being embedded in paraffin, sections of a 10 μm thickness were cut 1.5 mm anterior to the bregma, 1.0 mm anterior to the bregma, at the bregma, and 2.0 mm posterior to the bregma and were incubated in 0.3% hydrogen peroxide for 30 min and then incubated in blocking serum. The samples were incubated with primary antibodies at 4 °C overnight, followed by secondary antibody and ABC solution (#PK6101, PK6102) (Vector Laboratories, Inc., Newark, CA, USA), each for 1 h at room temperature. DAB tablets (D5905, Sigma-Aldrich, Burlington, MA, USA) were used for color development. High-resolution images of stained tissue sections were captured using a light microscope (Olympus BX53, Olympus, Tokyo, Japan) equipped with a digital camera (Olympus DP73, Olympus, Tokyo, Japan), ensuring consistent exposure settings across all slides. Images were captured at a 400× magnification, with 10 randomly selected sections in each target region and data collected from 5 to 7 animals per group. For immunofluorescence analysis, tissue sections were incubated with primary antibodies overnight at 4 °C, followed by incubation with Alexa-conjugated anti-rabbit or anti-mouse secondary antibodies at a dilution of 1:400. Confocal imaging was performed using the Zeiss LSM 880 system. Image processing was carried out using ImageJ (NIH), and the images were converted to grayscale or RGB format for the quantification of chromogen intensity. For DAB staining, the relevant color channels were split, with the brown DAB channel used for subsequent analysis. Thresholding was applied to separate positive staining from background noise, using automated thresholding provided by the software. The quantification of positive staining was performed by counting positively stained cells in 0.04 mm^2^. The primary antibodies used were anti-MAP2 antibody (17490-1-AP, 1:2000) (Proteintech, Rosemont, IL, USA), anti-GFAP antibody (16825-1-AP, 1:5000) (Proteintech, Rosemont, IL, USA), anti-Iba-1 antibody (10904-1-AP, 1:800) (Proteintech, Rosemont, IL, USA), anti-human alpha-synuclein antibody (syn211, ab80627, 1:2000) (Abcam, Cambridge, UK), and anti-phospho-alpha-synuclein antibody (pSyn#64, 1:1000) (Wako, Tokyo, Japan).

### 2.8. Electron Microscopical Studies

After collecting EVs through ultracentrifugation, EV pellets were fixed with 2.5% glutaraldehyde in PBS (pH 7.4) for 1 h. After post-fixation with 1% osmium tetroxide in PBS, the EVs were dehydrated through a graded ethanol series from 50% to 70% and stained with 1% uranyl acetate in 70% ethanol. The EVs were further dehydrated with 100% ethanol and embedded in LX112 resin (Ladd) and polymerized. The resin blocks were sectioned to a thickness of 70 nm. Transmission electron microscopy analysis was performed using the Hitachi HT7700 transmission electron microscope.

### 2.9. RNA Extraction and Real-Time Quantitative PCR

Total RNA was extracted from mouse brains using RNeasy Mini (Qiagen, Venlo, The Netherlands) with DNase treatment (Qiagen, Venlo, The Netherlands). Reverse transcription was performed using SuperScript III Reverse Transcriptase (ThermoFisher, Waltham, MA, USA). Real-time PCR to analyze human ATP13A2 expression was conducted using a TaqMan Fast Advanced MasterMix (ThermoFisher). Signals were recorded and analyzed by the QuantStudio 3 Real-Time PCR System (Applied Biosystems, Waltham, MA, USA). β-Actin was used as an internal control.

### 2.10. Western Blotting

Immunoblotting was conducted as described previously [8,23]. Protein lysates from mouse brains were prepared following the method in [19]. A total of 20 µg of lysate was mixed with 4× Laemmli buffer, boiled for 10 min, and resolved on a 4–20% acrylamide gel (Invitrogen). Proteins were then transferred onto PVDF membranes (Millipore, Burlington, MA, USA) and blocked with 3% PBS containing 0.1% Tween-20 (PBS-T) at room temperature for 1 h. The membranes were incubated overnight at 4 °C with anti-human Vimentin (AB_10897167, BD Biosciences, 1:1000) and anti-human alpha-synuclein (C-20, 1:1000) (Santa Cruz, Dallas, TX, USA) in PBS-T supplemented with 3% BSA. The detection of the primary antibodies was achieved using horseradish peroxidase-conjugated anti-mouse IgG (Santa Cruz, 1:10,000) and Enhanced Chemiluminescence (Amersham, Chicago, IL, USA) and visualized using the FUSION FX imaging system.

### 2.11. Cell Culture

Human induced pluripotent stem (iPS) cells were reprogrammed as described in a previous study [24]. Two control iPSCs (Cont 1 and 3) and two ATP13A2 mutant iPSCs (Mut 1 and 2) were characterized in earlier reports [25,26]. All iPSCs were cultured on irradiated mouse embryonic fibroblasts (MEFs) in iPS cell media containing DMEM/F12 (STEMCELL Technologies, Vancouver, BC, Canada), 20% knockout serum replacement (Invitrogen, Waltham, MA, USA), L-glutamine, nonessential amino acids, 2-mercaptoethanol (Invitrogen), 10 ng/mL FGF-Basic (AA1-155) recombinant human protein (Invitrogen), and penicillin/streptomycin at 37 °C in 5% CO_2_. Differentiation into dopaminergic (DA) neurons was performed following the protocol outlined in a previous publication [24]. We used DA neurons that had been differentiated for 40 days. Differentiation toward astrocytes and maturation was conducted as described previously [27]. The astrocytes that had been differentiated for 35 days were used for the experiments. Primary microglia (HMC3 cell line) were obtained from the American Type Culture Collection (ACTT) (CRL-3304) (ACTT, Manassas, VA, USA) and cultured according to the manufacture’s protocol. The microglia were infected with either scrambled shRNA or human TAP13A2 shRNA at a MOI of 1 and were used for the experiment 3 days post-infection (DPI).

### 2.12. Immunocytochemistry

Immunocytochemical analysis was performed as previously described [8,23]. Briefly, after fixation in 4% paraformaldehyde, dopaminergic (DA) neurons, astrocytes, and microglia were permeabilized and blocked in PBS containing 0.1% saponin, 1% BSA, and 5% normal goat serum for 20 min. The following primary antibodies were used: anti β-iii-tubulin (Covance, #MMS-435P, 1:1000 or Covance, #MRB-435P, 1:1000), tyrosine hydroxylase (EMD Millipore, #657012, 1:1000), anti-GFAP antibody (16825-1-AP, 1:5000) (Proteintech, Rosemont, IL, USA), and anti-Iba-1 antibody (10904-1-AP, 1:800) (Proteintech, Rosemont, IL, USA). The specimens were incubated overnight, washed three times in PBS, and then incubated with Alexa-conjugated anti-rabbit or anti-mouse secondary antibodies (1:400). Confocal imaging was carried out using the Zeiss LSM 880 confocal system (Zeiss, Oberkochen, Germany).

### 2.13. Statistical Analysis

All data were prepared for analysis with standard spreadsheet software (Microsoft Excel version 16.90.2). The Shapiro–Wilk test was employed to assess whether the sample data followed a normal distribution. Then, statistical analysis was performed by the Student *t*-test and one-way ANOVA followed by Tukey’s multiple comparisons test, depending on the experiments. All error bars represent SEM in the figures.

## 3. Results

### 3.1. Alternation of EV Secretion Affected A-Syn Accumulation and Propagation in Mouse Brains

To analyze the effect of EVs on α-syn propagation in mouse brains, we utilized *Atp13a2*-null mice where the *Atp13a2* gene was genetically deleted [19]. Following the established protocol, we isolated EVs from mouse brains (Figure 1A) and quantified them using NTA [8,20] (Figure 1B). We found that the number of EVs decreased in *Atp13a2*-null mouse brains (Figure 1C). We prepared human α-syn PFFs with a mean length of 22.4 ± 10.4 nm (mean ± SD) (Figure 1D). Subsequently, we inoculated α-syn PFFs into the right STR as described previously [20] (Figure 1E). Three months after inoculation, we conducted IHC analysis. Brain sections were prepared 1.5 mm from the bregma (most frontal), 1.0 mm from the bregma, 0 mm from the bregma (at the injection site), and −2.0 mm from the bregma (Figure 1F). We counted Lewy body (LB)- and neurite (LN)-like inclusions that were positive for the anti-human α-syn antibody syn211. Our analysis revealed no difference in α-syn PFF propagation between wild-type (WT) and *Atp13a2*-null mice (Figure 1F,G). Then, we attempted to increase EV secretion through lentivirus-mediated ATP13A2 overexpression. One week after injecting lentivirus carrying human ATP13A2 (LV-ATP13A2) or lentivirus carrying an empty vector (LV-EV) as a control in the right STR, we quantified ATP13A2 expression levels and the number of EVs. We confirmed human ATP13A2 expression (Figure 1I). EVs were isolated from mouse brains (Figure 1J) and quantified using NTA (Figure 1K), revealing an increase in EVs through ATP13A2 overexpression (Figure 1L). One week after injecting LV-ATP13A2 or LV-EV in the right STR, we administered α-syn PFFs in the same region. Three months later, the mice were sacrificed and IHC analysis was performed (Figure 1M,N). We found that the overexpression of ATP13A2 decreased regional α-syn PFF accumulation, as evidenced in sections 0.0 mm from the bregma (*p* = 0.005 for LBs and LNs). Additionally, we observed distant propagation, as shown in the sections 1.0 and 1.5 mm from the bregma (*p* = 0.001 for LBs and LNs) and sections −2.0 mm from the bregma (*p* = 0.001 for LBs and LNs) (Figure 1M,N). We confirmed α-syn PFF levels through immunoblotting (Figure 1O). These data suggest that, contrary to expectations, enhanced EV secretion mediated by ATP13A2 overexpression attenuated both the regional and distant propagation of α-syn PFFs.

### 3.2. Inoculated A-Syn PFFs Were Preferentially Taken up by Glial Cells

Recent studies have demonstrated that after α-syn PFFs are taken up by microglia, they became encapsulated within EVs and are subsequently released, eventually being taken up by neurons [28,29]. To investigate which type of cells take up more α-syn PFFs, we analyzed the mouse brains at various early time points following inoculation. The mice were perfused at 1, 3 and 6 h, as well as 1 day, 1 week, 3 weeks, and 3 months, post-inoculation (Figure 2A). The results of IHC revealed that α-syn PFFs increased until 3 h after injection and then decreased until 3 days and then increased again until 3 weeks at the injection site (Figure 2B, right STR). In contrast, α-syn PFFs were invisible until 6 h after injection, followed by a gradual increase until 3 weeks on the left side, opposite to the injection site (Figure 2C, left STR). Similarly, α-syn PFFs were not detected until 1 day after injection, after which they gradually increased until 3 weeks 1.5 mm from the bregma in the left cortex (Figure 2D). Then, we analyzed the types of cells that took up α-syn PFFs in the right STR. We found that they were initially taken up by microglia, followed by astrocytes, and these α-syn PFFs disappeared shortly afterward (Figure 2E,F). Neurons, on the other hand, exhibited a slower but more consistent uptake of α-syn PFFs, ultimately reaching their highest concentration at 3 weeks. Interestingly, ATP13A2 overexpression reduced the uptake of α-syn PFFs by neurons (Figure 2G). Notably, in both regions close to and distant from the injection site, neurons were the primary cells that took up α-syn PFFs (Figure 2H, left STR; I, left cortex).

These results demonstrate that the accumulation of α-syn PFFs in glial cells precedes that in neurons and that increased ATP13A2 expression attenuates the accumulation of α-syn PFFs in neurons.

### 3.3. Phosphorylated A-Syn PFFs Were Predominantly Accumulated in Neurons

**Figure 3:**
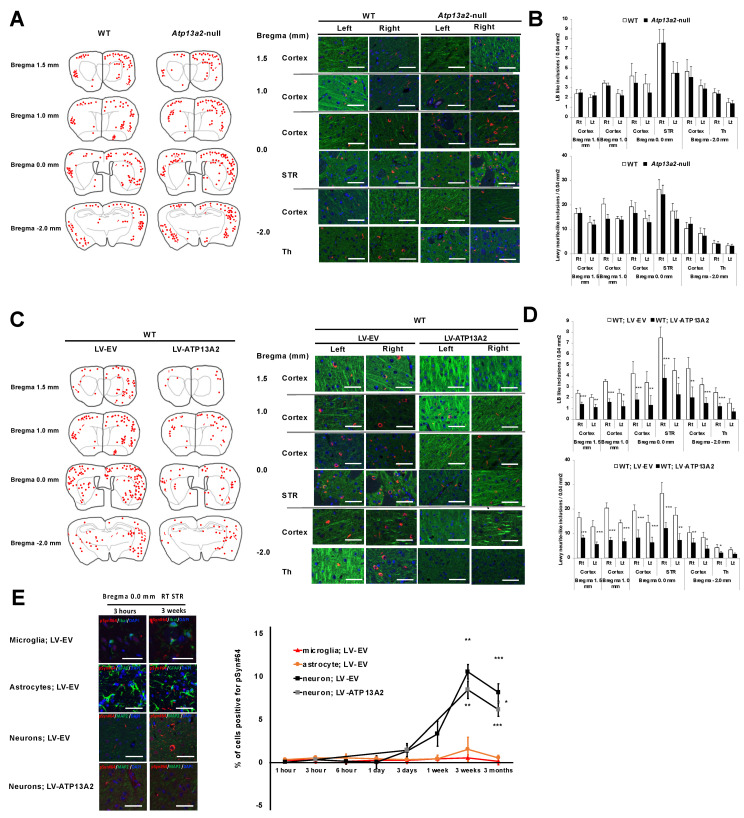
Phosphorylated α-syn PFFs were predominantly accumulated in neurons. (**A**) Left: distribution of LB- (red circle) and LN- (red line) (red line) like inclusions that were positive for the phosphorylated α-syn antibody in WT and *Atp13a2*-null mice three months after human PFF inoculation. The four sections were cut 1.5 mm anterior to the bregma (top), 1.0 mm anterior to the bregma (second from the top), at the bregma (second from the bottom), and 2.0 mm posterior to the bregma (bottom). Double immunofluorescence staining was performed with phosphor-α-syn (red) and MAP2 (green), as well as DAPI (blue), for nuclear staining. Right: representative images of each region from the WT and *Atp13a2*-null mice three months after mouse PFF inoculation. (**B**) Quantitative analysis of LB- (red circle) and LN- (red line) (red line) like inclusions in each section (n = 5–7). (**C**) Left: distribution of LB- (red circle) and LN- (red line) like inclusions that were positive for the phosphorylated α-syn antibody in the WT; LV-EV and WT; LV-ATP13A2 mice three months after human PFF inoculation. Right: representative images of each region from WT; LV-EV and WT; LV-ATP13A2 mice, in which each lentivirus was injected one week prior to α-syn PFF inoculation. Double immunofluorescence staining was performed with phospho-α-syn (red) and MAP2 (green), as well as DAPI (blue), for nuclear staining. (**D**) Quantitative analysis of LB- (red circle) and LN- (red line) (red line) like inclusions in each section (n = 5–7; * *p* < 0.03; ** *p* < 0.01; *** *p* < 0.001). (**E**) Left: representative images from the section 0.0 mm from the bregma in the right STR 3 h (left) and 3 weeks (right) after inoculation. Double immunofluorescence staining was performed for PFFs (red), Iba-1 (green) for microglia, PFFs (red) and GFAP (green) for astrocytes, or PFFs (red) and MAP2 (green) for neurons. DAPI (blue) was used for nuclear staining. Right: quantitative analysis of phospho-α-syn-positive inclusions in each section (n = 3; * *p* = 0.03; ** *p* = 0.005; *** *p* = 0.001). Values are the mean ± SEM. Scale bars represent 100 µm for (**A**,**C**,**E**). The mean and SD were calculated from 5 to 7 mice per group. Data were analyzed by the Student *t*-test (n = 5, (**B**,**D**)) or one-way ANOVA followed by Tukey’s multiple comparisons test (n = 5–7, (**E**)).

The propagated α-syn eventually accumulated in a phosphorylated form, detectable by an anti-phosphorylated α-syn antibody in Lewy bodies or Lewy neurites [2,30,31]. Three months after inoculation, we performed IHC analysis and found no difference in the accumulation of phospho-α-syn between WT and *Atp13a2*-null mice (Figure 3A,B). We then attempted to increase EV secretion through lentivirus-mediated ATP13A2 overexpression. One week after injecting LV-EV or LV-ATP13A2 into the right STR, we injected α-syn PFFs into the same region. Three months after, we sacrificed the mice and conducted IHC analysis (Figure 3C,D). We found that increased EV secretion decreased the regional accumulation of phosphorylated α-syn, as shown in sections 0.0 mm from the bregma (*p* = 0.03 for LBs and LNs). Additionally, we observed reduced distant α-syn PFF propagation, as shown in sections 1.0 and 1.5 mm from the bregma (*p* = 0.001 for LBs and LNs) and sections −2.0 mm from the bregma (*p* = 0.001 for LBs and LNs) (Figure 3C,D). Then, we analyzed the types of cells that accumulated phospho-α-syn. To this end, we performed double immunofluorescence analysis using antibodies against the anti-phospho α-syn antibody with MAP2 for neurons, GFAP for astrocytes, and Iba-1 for microglia in WT mice. We found that they were predominantly accumulated in neurons and were barely detected in microglia or astrocytes (Figure 3E). This neuronal accumulation was reduced by lentivirus-mediated ATP13A2 overexpression (Figure 3E). These results demonstrate that the depletion of Atp13a2 did not, but ATP13A2 overexpression did, reduce the accumulation of phospho-α-syn, which predominantly occurred in neurons.

### 3.4. Glial Cells Contributed to A-Syn Propagation in DA Neurons Through EV Secretion

**Figure 4:**
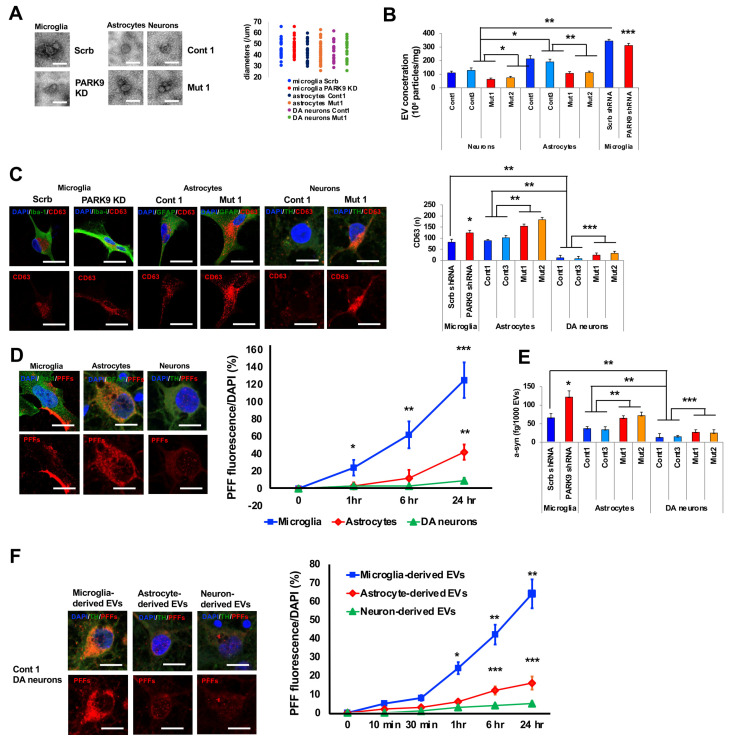
Glial cells contributed to α-syn propagation in DA neurons through EV secretion. (**A**) Left: electron microscopic analysis of EVs isolated from cultured primary microglia, iPSC-derived astrocytes, and DA neurons. In primary microglia, ATP13A2 expression was altered through shRNA-mediated knockdown. Right: analysis of the size of each vesicle. (**B**) Quantitative analysis of EVs isolated from primary microglia, iPSC-derived astrocytes, and DA neurons. (n = 6; * *p* = 0.003; ** *p* = 0.001; *** *p* = 0.01). (**C**) Left: representative images of CD63-positive vesicles in primary microglia, iPSC-derived astrocytes, and DA neurons. Double immunofluorescence staining was performed with CD63 (red) and Iba-1 (green) for microglia, CD63 (red) and GFAP (green) for astrocytes, or CD63 (red) and TH (green) for DA neurons. DAPI (blue) was used for nuclear staining. Right: quantitative analysis of CD63-positive vesicles in each cell (n = 6–10; * *p* = 0.001; ** *p* = 0.003; *** *p* = 0.03). (**D**) Left: representative images of α-syn PFF uptake in primary microglia, iPSC-derived astrocytes, and DA neurons 24 h post-α-syn PFF exposure. Double immunofluorescence staining was performed using PFFs (red) and Iba-1 (green) for microglia, PFFs (red) and GFAP (green) for astrocytes, or PFFs (red) and TH (green) for DA neurons. DAPI (blue) was used for nuclear staining. PFFs were stained with an anti-α-syn antibody (syn211). Right: quantitative analysis of changes in PFF fluorescence (red) in primary microglia, iPSC-derived astrocytes, and DA neurons at each time point (n = 6; * *p* = 0.01; ** *p* = 0.003; *** *p* = 0.001). (**E**) Quantitative analysis of fibrillar/oligomer forms of α-syn in EVs isolated from the media culturing primary microglia, iPSC-derived astrocytes, and DA neurons 24 h after α-syn PFF exposure (n = 3; * *p* = 0.001; ** *p* = 0.01; *** *p* = 0.007). (**F**) Left: representative images of DA neurons showing the uptake of α-syn PFFs encapsulated in EVs derived from primary microglia (microglia-derived EVs), iPSC-derived astrocytes (astrocyte-derived EVs), and DA neurons (neuron-derived EVs) 24 h after exposure. Double immunofluorescence staining was performed using antibodies against PFFs (red) and TH (green), as well as DAPI (blue), for nuclear staining. Right: quantitative analysis of α-syn PFF uptake in DA neurons following exposure to microglia-derived EVs, astrocyte-derived EVs, and neuron-derived EVs at specified time points. (n = 3; * *p* = 0.01; ** *p* = 0.001; *** *p* = 0.03). Values are the mean ± SEM. Scale bars represent 100 nm for (**A**), 20 µm for the neurons in (**C**) and (**D**,**F**), and 50 μm for the microglia and astrocytes in (**C**). The mean and SD were calculated from triplicate samples from two independent experiments. Data were analyzed by the Student *t*-test (n = 10–20, (**B**,**C**,**E**), microglia) or one-way ANOVA followed by Tukey’s multiple comparisons test (n = 5–7, (**B**,**C**,**E**), neurons and astrocytes, (**D**,**F**)).

To further investigate the differential uptake of α-syn PFFs by neurons and glia, we utilized dopaminergic (DA) neurons and astrocytes that were differentiated from iPSCs derived from healthy controls and patients carrying *ATP13A2* mutations. We also used primary microglia in which *ATP13A2* expression was modulated by shRNA. Following the established protocols, we isolated EVs secreted from DA neurons and astrocytes, as well as primary microglia. First, we conducted morphological analysis of EVs and observed no significant differences in the size of EVs across the different cell types (Figure 4A). Then, we quantified the EVs released by each cell type and found that microglia released significantly more EVs compared to neurons and astrocytes and astrocytes released significantly more EVs compared to neurons (Figure 4B). Importantly, ATP13A2 deficiency significantly reduced the number of EVs from neurons, astrocytes, and microglia (Figure 4B). These EVs were generated as ILVs in MVEs, which were visualized with a CD63 antibody (Figure 4C, bottom). We found that microglia and astrocytes contained MVEs more than neurons, and ATP13A2-deficiencies resulted in increased MVEs in these cells (Figure 4C, right). To analyze α-syn PFF uptake, we added α-syn PFFs to the culturing media for 24 h and isolated EVs containing these PFFs from the media. We utilized ELISA that could specifically quantify α-syn fibrils/oligomers [20]. The results revealed that α-syn PFFs were taken up by glial cells much faster and to a greater extent than by DA neurons (Figure 4D). Importantly, ATP13A2 knockdown in microglia, as well as deficiencies in neurons and astrocytes, resulted in an increased amount of α-syn PFFs per EV (Figure 4E). Finally, we analyzed whether the uptake of EVs was influenced by the types of cells that released them. To this end, we exposed DA neurons and astrocytes, as well as primary microglia, to α-syn PFFs and then isolated EVs containing these PFFs. Then, we exposed these α-syn PFF-encapsulated EVs to DA neurons. We found that DA neurons took up α-syn PFFs in glial EVs more rapidly and in greater amounts than from neuronal EVs (Figure 4F). As a result, α-syn PFFs accumulated more in neurons (Figure 4F).

Taken together, these findings suggest that glial cells rapidly uptake α-syn PFFs and release them within EVs, which are subsequently internalized by neurons, underscoring the critical role of glial cells in facilitating the propagation of α-syn PFFs in neurons.

## 4. Discussion

While the depletion of *Atp13a2* in the mouse brains did not alter the propagation of α-syn PFFs (Figure 1F,G), consistent with a previous report [32], increased ATP13A2 levels decreased α-syn PFF propagation in the brains (Figure 1M,N), at least partially, by enhancing the secretary pathway associated with EVs. Recent studies strongly suggest that α-syn transfers between organs, tissues, and neurons [33]. Initially, α-syn is transported along neuronal axons, with Lewy bodies and Lewy neurites—structures that accumulate α-syn—primarily found in neurons [30,31]. Postmortem examinations suggest that Lewy pathology progresses from the brainstem to the STR and subsequently to the cortex [2,30]. Additionally, some experimental studies support the hypothesis that Lewy pathology progresses from the gastrointestinal tract to the CNS [3,4]. More recently, α-syn and tau pathology have been shown to spread from the gut to the brain, at least partially, via the vagus nerve, corroborating the concept of transneuronal spreading [34]. However, other experimental studies have not fully replicated these findings [5]. For instance, α-syn PFFs inoculated in the gastrointestinal tract spread through the vagus nerve and form Lewy body-like aggregates in the brainstem, but they do not spread beyond the brainstem, highlighting the limitations of transneuronal propagation [5]. When α-syn PFFs were directly inoculated into the STR, Lewy pathology developed throughout the entire brain because the STR directly connects to many brain regions. In contrast, the inoculation of α-syn PFFs in the olfactory bulb resulted in only partial propagation, as the α-syn PFFs traveled to the neurons only two synapses away since the primary projection of the olfactory bulb is only to the amygdala [35]. These findings underscore the limitations of the transneuronal transmission of α-syn PFFs and suggest an alternative pathway because the olfactory bulb is considered one of the initial sites of α-syn propagation. Since EVs contain α-syn and can transport intracellular α-syn between neurons [20], patient-derived EVs are potentially used as a biomarker for PD. We have reported that EVs isolated from the serum of patients with PD and REM sleep behavior disorder (RBD) contain higher levels of filamentous α-synuclein compared to healthy individuals and patients with atypical parkinsonism [20]. Additionally, EVs isolated from the blood of PD and RBD patients exhibit greater seeding activity [20], suggesting a significant role in PD pathogenesis. Our study further demonstrated that EVs played a significant role in α-syn propagation within the mouse brains. Notably, contrary to the expectation, enhanced EV secretion did not lead to increased α-syn PFF propagation throughout the brain but rather decreased it (Figure 1 and Figure 3). Indeed, recent studies suggest that patient-derived exosomes can present or worsen α-syn pathology in mice [36,37]. However, our results indicate that EVs could alleviate the propagation of PFFs. This was likely due to our enhancement of endogenous EV secretion, allowing secreted α-syn PFFs to be taken up by regional glial cells and degraded, thereby preventing widespread propagation throughout the brain.

Our results also revealed the contribution of glial cells in α-syn PFF propagation in mouse brains (Figure 2F). The inoculated α-syn PFFs were primarily taken up by microglia and astrocytes (Figure 4D) and then were secreted, at least partially, in EVs (Figure 4E), which were more efficiently taken up by neurons compared to neuron-derived EVs (Figure 4F). Recent evidence increasingly support the significant role of microglia-derived EVs in α-syn propagation in mouse brains [28,29]. Depleting microglia stops propagation, indicating an important role of microglia in α-syn neuronal propagation [38]. Our investigation partially corroborates microglia’s role. Our observation revealed that α-syn PFFs were preferentially taken up by microglia and astrocytes, likely due to their phagocytotic ability (Figure 4). Given that the number of microglia and astrocytes exceeded that of neurons, and that these glial cells secreted significantly more EVs per cell (Figure 4B), glial EVs were consequently much more abundant than neuronal EVs in the brains. Recent studies have shown that EVs can selectively target recipient cells based on surface markers, with microglial markers playing a crucial role in neuronal uptake [29]. However, our results also revealed that enhanced EV secretion attenuated the propagation of α-syn PFFs, suggesting that EV secretion can be protective if glial cells stay healthy. Taken together, these findings suggest that glial EVs play a critical role for α-syn propagation in mouse brains.

Our data also demonstrated that, in contrast to the uptake of inoculated α-syn PFFs, which were preferentially taken up in glial cells rather than neurons, phosphorylated α-syn was exclusively accumulated only in neurons (Figure 3), consistent with previous pathological examinations [39]. This may have been partially due to the fact that microglia and astrocytes could efficiently secrete internalized α-syn PFFs, leaving little behind in the cells (Figure 2F). In contrast, neurons were less efficient in secreting internalized α-syn PFFs, which meant that although, initially, uptake was minimal, α-syn PFFs persisted in the neurons over time, leading to the formation of Lewy bodies/neurites of which the main component was phosphorylated α-syn.

## 5. Conclusions

Our results reveal that glial EVs play a significant role in α-syn propagation. Interestingly, enhanced EV secretion does not facilitate, but rather partially impairs, α-syn propagation throughout the brain. Consequently, targeting EVs would be a novel strategy not only for synucleinopathies, including PD, but also for neurodegenerative disorders characterized by the propagation of misfolded proteins in the brain.

## Data Availability

The data supporting the reported results can be provided upon request.
